# Cæsarean Operation Performed Four Times in the Same Subject

**Published:** 1871-03

**Authors:** 


CÆSAREAN OPERATION PERFORMED FOUR TIMES IN THE SAME SUBJECT.—Dr. Oettler reports a curious case of this. The subject of it had a narrow pelvis, resulting from rickets, with which she had been affected from infancy. The first labor was in 1853, when she was 23 years of age, and her subsequent ones in 1857, 1858, 1859, and 1863. Each time she was delivered by Cæsarean section of a living and viable child, rapidly recovered from each operation, and at the date of the report was in good health. —*Revue de Therapeutique*, Sept. 15, 1870.

